# Supplementary material for: Mapping Multiplex Hubs in Human Functional Brain Networks
Source: Front Neurosci. 2016 Jul 15;10:326. doi: 10.3389/fnins.2016.00326 (PMC4945645; doi:10.3389/fnins.2016.00326)
Supplement: Supplementary file 1 [file Presentation1.PDF]

Supplementary Material
Mapping multiplex hubs in human functional brain network

Manlio De Domenico[1,*], Shuntaro Sasai[2,*], Alex Arenas[1]

[1] *Departament d'Enginyeria Informàtica i Matemàtiques, Universitat Rovira i Virgili, 43007 Tarragona, Spain*

[2] *Department of Psychiatry, University of Wisconsin - Madison, Madison, WI, USA*

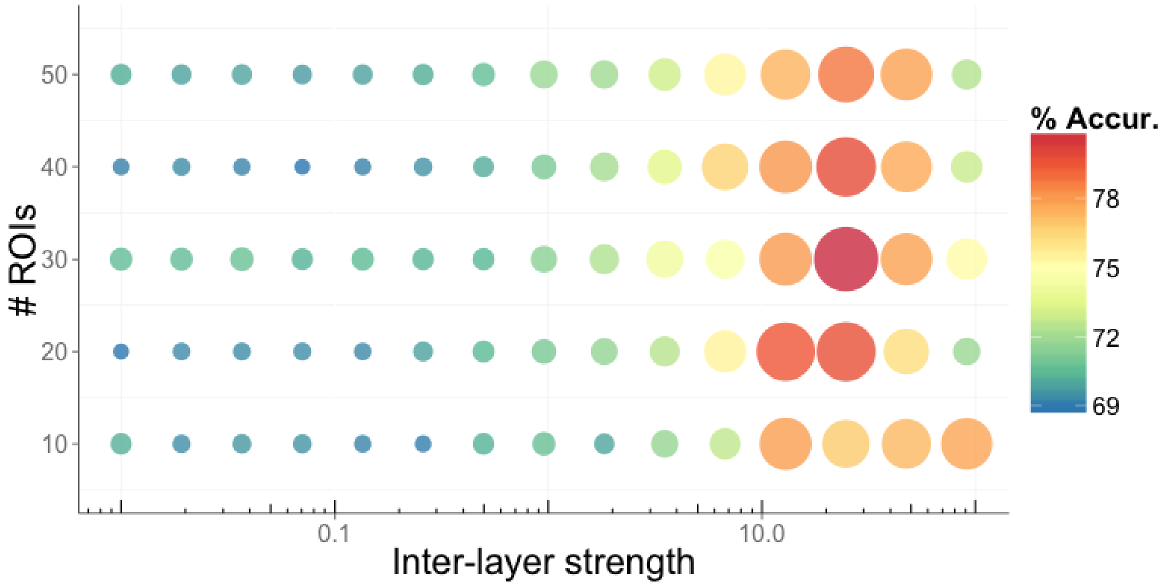

Supplementary Figure 1: **Maximizing classification accuracy.** Layers in the multiplex functional network are interconnected to allow the calculation of layer's and node's properties. However, the weight of such inter-layer links can not be deduced from the data. At the same time, not all ROIs have enough discriminant power and using sub-sets of them reduces the noise and generally improves the classification. We have chosen the value of the inter-layer strength ($D = 24.7708$) and the size of an appropriate sub-set of ROIs (30) where maximum classification accuracy is achieved (see Methods for details).

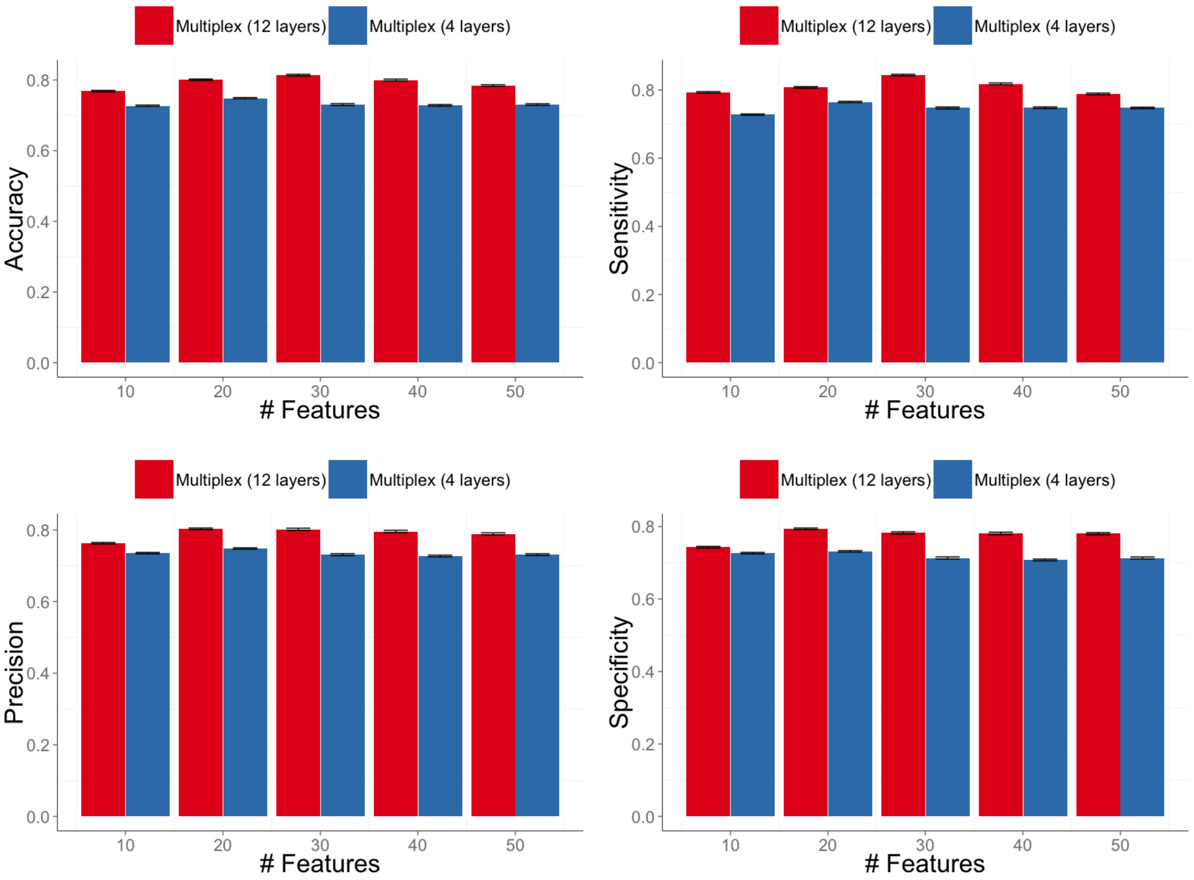

Supplementary Figure 2: **Discrimination performance of two different multiplex functional networks.** A new multiplex network, consisting only of the layers corresponding to the typical frequency range, has been built and used for discriminating between control and patient groups. The statistical indicators of the discrimination are compared against the full multiplex functional network discussed in the text, providing better overall discrimination. Bars indicate standard errors.

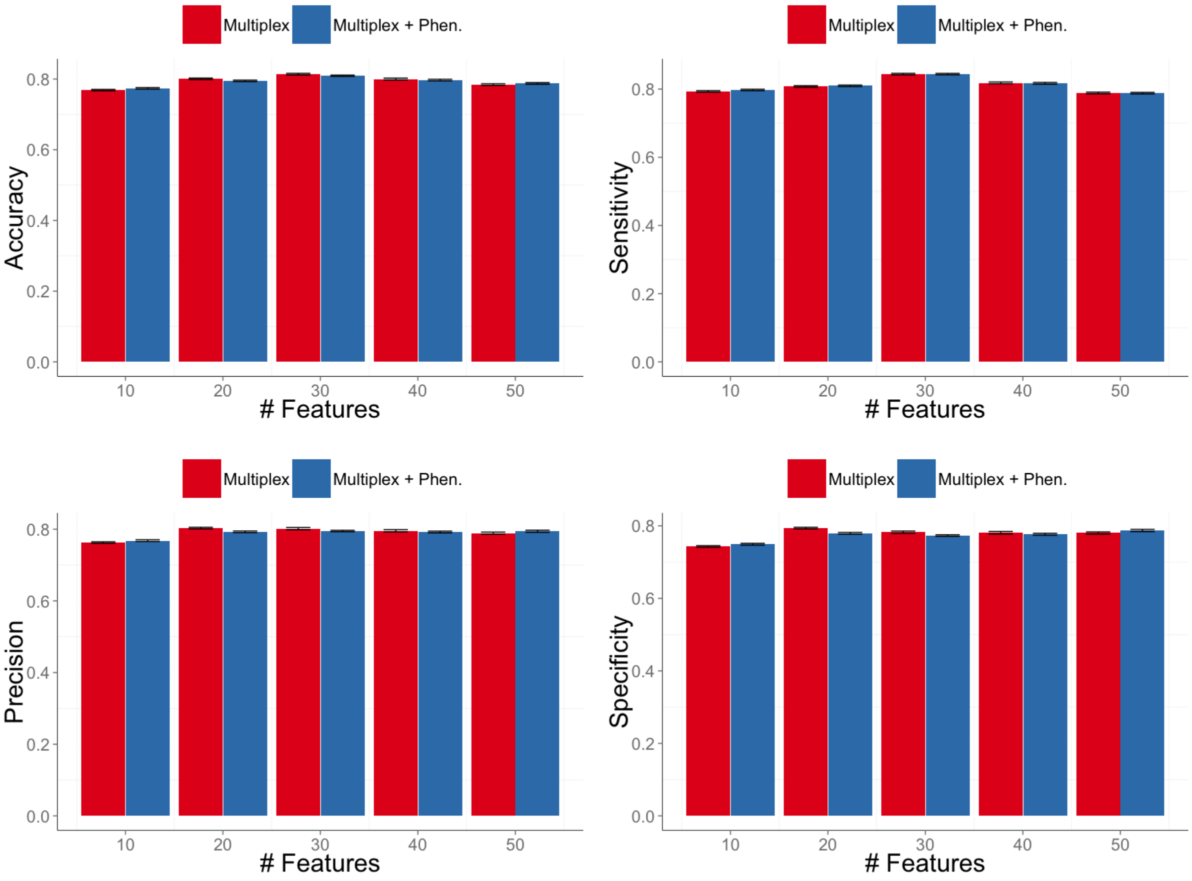

Supplementary Figure 3: **Discrimination performance of the multiplex functional networks with and without phenotypic information.** The statistical indicators of the discrimination between control and patient groups obtained from the full multiplex functional network before and after including phenotypic information in the machine learning process. The results are not significantly different, indicating that phenotypic data is redundant in this case and does not improve discrimination. Bars indicate standard errors.

Supplementary Table 1: Anatomical information on the 30 ROIs used for classification.

**Network model**	**Group**	**MNI coordinate**			**Talairach coordinate**			**Anatomical label**
		**X**	**Y**	**Z**	**X**	**Y**	**Z**	
multiplex	healthy	58	-16	7	53	-17	9	Transverse Temporal Gyrus
		32	-26	13	29	-27	14	Claustrum
		-3	42	16	-4	37	21	Anterior Cingulate
		0	30	27	-1	25	30	Cingulate Gyrus
		34	16	-8	31	14	-2	Claustrum
		-28	52	21	-27	46	26	Superior Frontal Gyrus
		32	14	56	29	7	55	Middle Frontal Gyrus
		-11	45	8	-11	40	14	Medial Frontal Gyrus
		-13	-17	75	-14	-23	69	Precentral Gyrus
	schizophrenia	-16	-5	71	-17	-11	67	Superior Frontal Gyrus
		24	-87	24	21	-85	19	Cuneus
		19	-8	64	16	-14	61	Superior Frontal Gyrus
		-3	26	44	-4	20	45	Medial Frontal Gyrus
		46	-59	4	42	-57	3	Middle Temporal Gyrus
		-7	-21	65	-8	-26	60	Medial Frontal Gyrus
		34	38	-12	31	35	-4	Inferior Frontal Gyrus
		-47	-76	-10	-45	-72	-12	Fusiform Gyrus
		-37	-29	-26	-36	-27	-22	Parahippocampal Gyrus
	common	11	-54	17	9	-53	15	Posterior Cingulate
		17	-80	-34	15	-74	-33	Uvula
		-50	-7	-39	-48	-5	-32	Inferior Temporal Gyrus
		-32	-1	54	-31	-6	52	Precentral Gyrus
full-band	healthy	-60	-25	14	-57	-26	14	Superior Temporal Gyrus
		-7	-52	61	-8	-55	54	Precuneus
		58	-16	7	53	-17	9	Transverse Temporal Gyrus
		6	-72	24	4	-71	20	Cuneus
		0	30	27	-1	25	30	Cingulate Gyrus
		-16	-77	34	-16	-76	28	Cuneus
		-3	-81	21	-4	-79	16	Cuneus
		10	-62	61	8	-64	54	Superior Parietal Lobule
	schizophrenia	-10	-18	7	-11	-19	8	Thalamus
		-20	64	19	-20	57	25	Superior Frontal Gyrus
		26	50	27	23	43	32	Superior Frontal Gyrus
		27	-97	-13	24	-92	-15	Fusiform Gyrus
		-55	-9	12	-53	-10	13	Precentral Gyrus
		36	22	3	33	19	8	Insula
		9	-4	6	7	-6	9	Thalamus
		47	-30	49	42	-33	46	Inferior Parietal Lobule
	common	0	-15	47	-1	-19	45	Paracentral Lobule
		-3	2	53	-4	-3	51	Medial Frontal Gyrus
		4	-48	51	2	-50	46	Precuneus
		-3	26	44	-4	20	45	Medial Frontal Gyrus
		-1	15	44	-2	10	44	Medial Frontal Gyrus
typical-band	healthy	58	-16	7	53	-17	9	Transverse Temporal Gyrus
		-60	-25	14	-57	-26	14	Superior Temporal Gyrus
		6	-72	24	4	-71	20	Cuneus
		11	-39	50	9	-42	46	Precuneus
		10	-62	61	8	-64	54	Superior Parietal Lobule
		8	-72	11	6	-70	8	Cuneus
		32	-26	13	29	-27	14	Claustrum
	schizophrenia	-10	-18	7	-11	-19	8	Thalamus
		-5	18	34	-6	13	35	Cingulate Gyrus
		26	50	27	23	43	32	Superior Frontal Gyrus
		7	8	51	5	2	50	Medial Frontal Gyrus
		5	23	37	3	17	39	Cingulate Gyrus
		0	30	27	-1	25	30	Cingulate Gyrus
		-55	-9	12	-53	-10	13	Precentral Gyrus
	common	-7	-52	61	-8	-55	54	Precuneus
		0	-15	47	-1	-19	45	Paracentral Lobule
		-3	2	53	-4	-3	51	Medial Frontal Gyrus
		4	-48	51	2	-50	46	Precuneus
		-3	26	44	-4	20	45	Medial Frontal Gyrus
		-1	15	44	-2	10	44	Medial Frontal Gyrus

Supplementary Table 2: Anatomical information on the ROIs identified as hubs.

MNI coordinate			Talairach coordinate			Anatomical label
X	Y	Z	X	Y	Z	
27	-97	-13	24	-92	-15	Fusiform Gyrus
-10	-18	7	-11	-19	8	Thalamus
52	-34	-27	48	-32	-23	Fusiform Gyrus
36	22	3	33	19	8	Insula
-25	-98	-12	-25	-92	-15	Fusiform Gyrus
65	-24	-19	60	-23	-15	Middle Temporal Gyrus
-40	-19	54	-39	-23	50	Postcentral Gyrus
6	-24	0	5	-24	2	Thalamus
33	-12	-34	30	-10	-27	Uncus
24	32	-18	22	30	-10	Sub-Gyral
34	54	-13	31	50	-4	Middle Frontal Gyrus
55	-31	-17	50	-30	-13	Inferior Temporal Gyrus
0	-15	47	-1	-19	45	Paracentral Lobule
66	-8	25	61	-11	26	Precentral Gyrus
-45	0	9	-43	-2	11	Insula
-56	-45	-24	-53	-42	-22	Fusiform Gyrus
-55	-9	12	-53	-10	13	Precentral Gyrus
25	-58	60	22	-60	53	Superior Parietal Lobule
-21	41	-20	-20	39	-11	Middle Frontal Gyrus
-31	-10	-36	-30	-8	-30	Uncus
9	54	3	8	49	10	Medial Frontal Gyrus
13	55	38	11	47	42	Superior Frontal Gyrus
-20	64	19	-20	57	25	Superior Frontal Gyrus
35	-67	-34	32	-62	-32	Cerebellar Tonsil
49	-3	-38	45	-2	-30	Middle Temporal Gyrus
-37	-29	-26	-36	-27	-22	Parahippocampal Gyrus
24	45	-15	22	42	-6	Medial Frontal Gyrus
-1	15	44	-2	10	44	Medial Frontal Gyrus
-18	63	-9	-18	59	0	Medial Frontal Gyrus
53	33	1	49	29	8	Inferior Frontal Gyrus

Supplementary Table 3: Statistical descriptors for age distribution in the two groups. The null hypothesis that phenotypic details in the two groups are sampled from continuous distributions with equal medians is not rejected for Age (P=0.4253) and Gender (P=0.1186), while it is rejected for Handed-ness (P=0.004).

	**# Subj.**	**Median**	**Avg**	**Std**	**L. quart.**	**H. quart.**	**# Females**	**# Left Hand.**
Healthy	74	34	35.8	11.6	26	44	23	1
Schizophrenia	71	37	38.1	14	25.25	50	14	10

Supplementary Table 4: Discriminating ROIs which are also hubs.

ROI	MNI coordinates			Talairach Daemon	
**Healthy**					
15	0	-15	47	Paracentral Lobule	Brodmann area 31
258	25	-58	60	Superior Parietal Lobule	Brodmann area 7
213	-1	15	44	Medial Frontal Gyrus	Brodmann area 6
**Schizophrenia**					
2	27	-97	-13	Fusiform Gyrus	Brodmann area 18
224	-10	-18	7	Thalamus	Medial Dorsal Nucleus
209	36	22	3	Insula	Brodmann area 13
1	-25	-98	-12	Inferior Occipital Gyrus	Brodmann area 17
247	33	-12	-34	Uncus	Brodmann area 20
11	55	-31	-17	Inferior Temporal Gyrus	Brodmann area 20
15	0	-15	47	Paracentral Lobule	Brodmann area 31
55	-45	0	9	Precentral Gyrus	Brodmann area 44
70	-55	-9	12	Precentral Gyrus	Brodmann area 43
102	13	55	38	Superior Frontal Gyrus	Brodmann area 9
114	-20	64	19	Middle Frontal Gyrus	Brodmann area 10
8	-37	-29	-26	Culmen	*
213	-1	15	44	Medial Frontal Gyrus	Brodmann area 6
